# Metabolic rewiring is associated with HPV-specific profiles in cervical cancer cell lines

**DOI:** 10.1038/s41598-021-96038-8

**Published:** 2021-09-06

**Authors:** Kalliopi I. Pappa, George Daskalakis, Nicholas P. Anagnou

**Affiliations:** 1 Cell and Gene Therapy Laboratory, Biomedical Research Foundation of the Academy of Athens (BRFAA), Athens, Greece; 2 First Department of Obstetrics and Gynecology, National and Kapodistrian University of Athens School of Medicine, Athens, Greece; 3 Laboratory of Biology, National and Kapodistrian University of Athens School of Medicine, Athens, Greece

**Keywords:** Cervical cancer, Metabolomics

## Abstract

Both HPV-positive and HPV-negative cervical cancers are associated with aberrant metabolism, although the oncogenic drivers remain elusive. Here we show the assessment of the metabolomic profiles of four distinct cervical cell lines, a normal and three cancer cell lines, one HPV-negative (C33A) and two HPV-positive (SiHa HPV16+, HeLa HPV18+), employing an ultra performance liquid chromatography and a high resolution mass spectrometry. Out of the total 462 metabolites, 248 to 326 exhibited statistically significant differences, while Random Forests analysis identified unique molecules for each cell line. The two HPV+ cell lines exhibited features of Warburg metabolism, consistent with the role of the HPV E6 protein. SiHa and HeLa cells displayed purine salvage pathway activity, while C33A cells revealed synthesis of cytidine, via a novel mechanism. These data document a highly dynamic HPV-specific rewiring of metabolic pathways occurring in cervical cancer. Therefore, this approach can eventually provide novel mechanistic insights into cervical carcinogenesis.

## Introduction

Systematic studies on several tumour types, have revealed a pattern of metabolic rewiring occurring during carcinogenesis, representing one of the hallmarks of cancer^1–3^. The particular metabolic deviations, reflect specific responses and a subsequent adaptation of the tumour cell to the particular acquired genetic and epigenetic aberrations generated both within the tumour cell populations and in the cellular microenvironment of the tumour. This metabolic response is tightly linked with cell proliferation and nutrient availability, stress signalling and regulation of energy homeostasis, coupled with external signalling events and the modification of particular metabolic pathways, such as carbohydrate metabolism and biosynthetic processes^1^. The resulting modified cancer metabolism provides the tumour cell with the flexibility and ability to acquire-under severe conditions, such as hypoxia, the appropriate and/or alternative nutrients necessary for its survival, the building of new biomass^2^, and the maintenance of the biochemical\homeostasis^3^. This network of aberrant metabolic reactions is closely integrated with major oncogenic pathways, mutational patterns and epigenetic regulators. These oncogene-driven alterations, affecting the activation of key transcription factors, lead to the deregulation of energy sensor pathways and cell cycle control, and result in autocrine and paracrine mechanisms, without compromising cell survival^4^.

A particular oncogenic mechanism leading to carcinogenesis and also to metabolic reprogramming, is the viral oncogenesis mediated by several human oncogenic viruses, which induce carcinogenesis in a multi-step process, by targeting crucial cellular pathways and key proteins, such as control of cell cycle progression, proliferation and evasion from cell death and eventually establishing a metabolic reprogramming pattern^5^. This particular reprogramming is induced either directly or indirectly by the viruses via modulation of upstream signalling pathways^5^. Human papilloma virus (HPV) represents one of the most potent oncogenic viruses, and is responsible for all cases of cervical cancer and for the majority of anal, vaginal, oral cavity and pharyngeal carcinomas. Specifically, the high-risk HPV types 16 and 18, exhibit a unique ability to induce cell proliferation in the basal and parabasal cell layers. By upregulating the expression of multiple viral gene products, especially E6 and E7 oncoproteins, these high-risk HPV types, can inhibit p53 and RB tumour suppressors, respectively, which can lead to the disruption of critical cellular regulatory pathways controlling cell cycle entry, differentiation, apoptosis, cell polarity^6^, and to the establishment of an aberrant cellular metabolism^5^.

It is of interest, that both HPV-positive and HPV-negative cervical cancers are associated with disruption of these same pathways^6,7^, however, our current understanding of the relative contribution of these oncogenic drivers in the development and progression of cervical cancer remains incomplete^6^.

Therefore, the goal of the current study was to systematically characterize for the first time, the metabolomic profiles of four informative cervical cell lines in order to identify specific metabolic derangements driving carcinogenesis, which can be attributed to the presence or absence of the initial HPV infection. To this end, we characterized the metabolomic profiles of four distinct and complementary informative cervical cell lines, i.e. a normal cervical keratinocyte (HCK1T) cell line^8,9^, and three cervical cancer cell lines, one HPV-negative (C33A), and two HPV-positive (SiHa HPV16+ and HeLa HPV18+). Specifically, HeLa cells have been derived from a patient with cervical adenocarcinoma and contain human papilloma virus 18 (HPV-18) sequences, while p53 expression has been reported to be low with normal levels of pRB^7^. The cells exhibit epithelial morphology and grow adherently, reproducing an entire generation about every 24 h. SiHa cells were established from a Japanese patient with squamous cell cervical carcinoma grade II, contain an integrated human papillomavirus type 16 genome (HPV-16, 1 -2 copies/cell), and can form poorly differentiated epidermoid carcinoma (grade III) in nude mice^7^. The C-33 A cell line is one of a series of lines (ATCC CRL-1594 and ATCC CRL-1595) derived by N. Auersperg from cervical cancer biopsies^7^. The line exhibits an adherent epithelial morphology, expressing high levels of p53, while pRB is present but abnormal in size. The cells are negative for human papillomavirus DNA and RNA. Lastly, HCK1T cells were recently established and immortalized from normal human cervical keratinocytes, by the introduction of the human catalytic subunit of telomerase reverse transcriptase (*hTERT*), and represent a reliable normal human cervical keratinocyte model, and subsequently a suitable in vitro model system for HPV-mediated multistep carcinogenesis^8,9^.

Employing an ultra performance liquid chromatography and high resolution-accurate mass spectrometry approach on these complementary cell lines, offers a reliable comparison of the metabolomics effects of the two most common high-risk HPV types versus the HPV-negative and normal cervical cells, and can eventually lead to the discovery of novel mechanisms of viral carcinogenesis.

## Results

### Principle component, hierarchical cluster and Random Forests analysis

Principal component analysis (PCA) was employed to determine whether the four cell lines can be segregated based on differences in their overall metabolic signature. Results revealed very strong segregation of each cell line, forming four tightly grouped clusters (Extended Data Fig. 1). Consistent with this, hierarchical cluster analysis (HCA) of the dataset revealed strong separation of each cell line, again segregating into four distinct groups (Extended Data Fig. 2). Furthermore, Random Forests analysis was employed to identify metabolites that differentiated among the four cell lines and contributed most strongly to the group binning, yielding a predictive accuracy of 100%, compared to 25% by random chance alone, as shown in Extended Data Fig. 3. Thus, these data documented that the biochemical differences among the samples of the four cell lines were highly pronounced. Collectively, the data confirmed that the four informative cells lines exhibit highly distinct global biochemical profiles.

**Figure 1:**
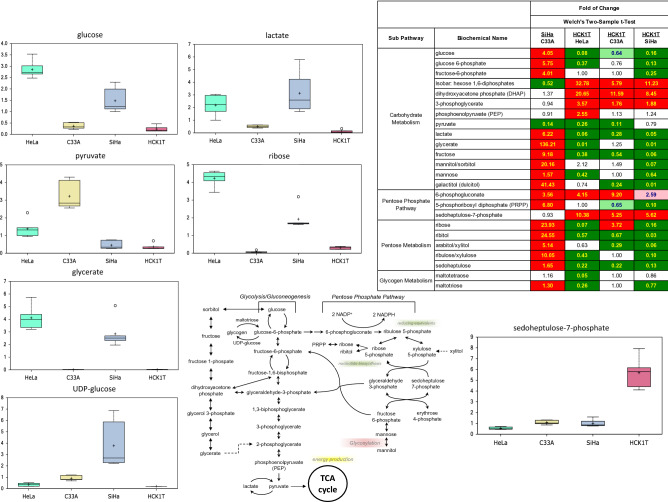
Metabolomic analysis of the altered carbohydrate metabolism of the four cervical cell lines. Selected biochemicals exhibiting salient changes among the four cell types are displayed as box plots. The relevant pathway map and a representative heat map is shown, including the major biochemical changes. Pathway schematics for Figs. 1, 2, 3, 4, 5 and 7, were prepared by GAM at Metabolon, Inc., utilizing established sources, including KEGG (https://www.genome.jp/kegg/) and published literature to confirm each biochemical step of the pathway. Green color indicates significant difference (*P* ≤ 0.05) between the groups shown, metabolite ratio of < 1.00; light green indicates narrowly missed statistical cut-off for significance 0.05 < *P* < 0.10, metabolite ratio of < 1.00; red indicates significant difference (*P* ≤ 0.05) between the groups shown, metabolite ratio of ≥ 1.00; light red indicates narrowly missed statistical cut-off for significance 0.05 < *P* < 0.10, metabolite ratio of ≥ 1.00, and non-coloured cells, indicates that mean values are not significantly different for that comparison. Each box plot provides a measure of scaled intensity, normalised by protein content. For further details see Extended Data Table 2.

**Figure 2:**
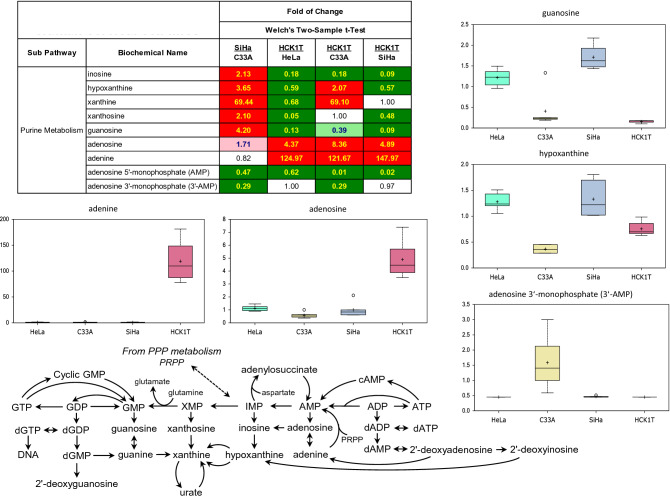
Metabolomic analysis of the differential nucleic acid metabolism of the four cervical cell lines. Selected biochemicals are displayed as box plots. Each box plot provides a measure of scaled intensity, normalised by protein content. The relevant pathway map and a heat map with the major biochemical changes is shown. For the color key of the heat map, see the legend of Fig. 1.

**Figure 3:**
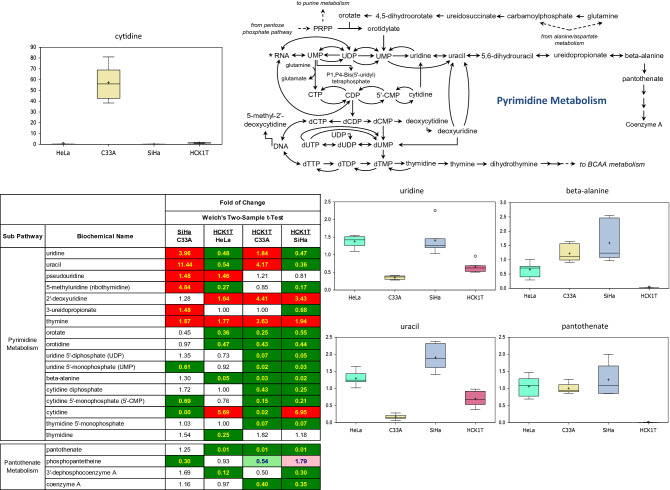
Metabolomic analysis of the pyrimidine metabolism of the four cervical cell lines. Levels of cytidine, uridine, uracil and other biochemicals displaying changes are shown as box plots. Each box plot provides a measure of scaled intensity, normalised by protein content. The pyrimidine pathway map and a heat map, with the major biochemical changes is also shown. For the color key of the heat map, see the legend of Fig. 1.

### Significantly altered biochemicals among the four cell lines

Following normalization to Bradford protein concentration, log transformation and imputation of any missing values with the minimum observed value for each compound, the Welch’s two-sample *t*-test was used to identify biochemicals that differed significantly between the four cell lines. A summary of the biochemicals that achieved statistical significance (*P* ≤ 0.05) from the six-pairs comparisons of cell lines, as well as those approaching significance (0.05 < *P* < 0.10), is shown in Extended Data Table 1 and 2. The dataset comprised a total of 462 biochemicals, 439 compounds of known identity, and 23 of unknown structural identity, the nature of which is under investigation.

### Altered carbohydrate metabolism

Transformed cells are characterized by cytoadaptation to optimize substrate availability for deregulated growth and biomass generation. Within this context, the four cell lines exhibited differential utilization of carbohydrates. Specifically, HPV-positive HeLa and SiHa cells, showed preferred accretion of glucose and glycolytic intermediates, such as glucose-6-phosphate, fructose-6-phosphate, and lactate (Fig. 1; heat map, box plots). These changes occur with relative concomitant depletion of pyruvate, consistent with activation of aerobic glycolysis (Warburg metabolism) in these cells. In support of this, we observed significant increases in glycerate levels, which also feed into pyruvate/lactate production via conversion to 2-phosphoglycerate (Fig. 1; pathway map). Both SiHa and HeLa cells also showed striking increases in ribose and ribitol (a pentose formed by the reduction of ribose) intermediates in the pentose phosphate pathway (PPP). SiHa cells are further differentiated by their ability to produce UDP-glucose, which may suggest at least partial shunting of carbons for glycogen utilization in these cells. Conversely, the HPV-negative HCK1T and C33A cells, broadly exhibited reduced levels of these intermediates, but higher levels of hexose 1,6-diphosphates (isobar), dihydroxy-acetone phosphate (DHAP), and 3-phosphoglycerate (Fig. 1; heat map). Of note, HCK1T cells preferentially exhibited enrichment in the PPP intermediates 6-phosphogluconate and sedoheptulose-7-phosphate, that may indicate alternate carbon utilization in these cells (Fig. 1; heat map). Together, these results highlight differences in cancer cell metabolism, with SiHa and HeLa cells displaying Warburg signatures, distinct from C33A and HCK1T cells that exhibited an atypical profile of intermediates associated with DHAP, probably as a mechanism to provide carbons for lipid synthesis.

### Cervical cancer cells exhibit differential nucleic acid metabolism

Nucleotide synthesis precursors are generated, in part, through the PPP, converting the glycolysis intermediate glucose-6-phosphate to 6-phosphogluconate and then to ribose-5-phosphate (Fig. 2; pathway map). The HPV-associated cells (SiHa and HeLa) both displayed significantly elevated levels of purine derivatives inosine and guanosine, as well as nucleotide breakdown products hypoxanthine, xanthine, and xanthosine (Fig. 2; heat map, box plots). Conversely, HCK1T cells exhibited elevations in adenosine metabolism (adenosine and adenine), which aside from nucleic acid synthesis, also provides intermediates for nicotinate adenine dinucleotide (NAD) and ATP production, and G-protein receptor signaling. The HPV-negative C33A cells, showed relative depletion in the levels of these metabolites, but instead displayed accretion in AMP metabolites adenosine 5'-monophosphate (5′-AMP) and adenosine 3′-monophosphate (3′-AMP), suggesting an energy-depleted state and/or elevated nucleic acid turnover, relative to other cell types (Fig. 2; heat map). Rather, these cells showed evidence of altered pyrimidine synthesis, which also derives from PPP metabolism (Fig. 3; pathway map, box plot). Specifically, C33A cells displayed unusually high concentrations of cytidine and cytidine 5′-monophosphate (5′-CMP) relative to other cells that exhibit differential availability of other pyrimidine intermediates, such as uridine and uracil (Fig. 3; heat map, box plots). Cytidine levels also support lipid biosynthesis through cytidine 5′-diphosphocholine production. Finally, HCK1T cells uniquely exhibited depleted levels of β-alanine, which aside of being a reporter for pyrimidine synthesis, also supplies pantothenate (vitamin B5) metabolism to drive Coenzyme A (CoA) synthesis (Fig. 3; pathway map). Given the direct correlation observed between low lactate and β-alanine levels, this may represent part of a yet uncharacterized metabolic signature in HCK1T cells. Taken together, each cell line showed evidence of preferential utilization of nucleotide intermediates, which results in distinct biochemical profiles.

### Cell lines display differential nicotinate metabolism

**Figure 4:**
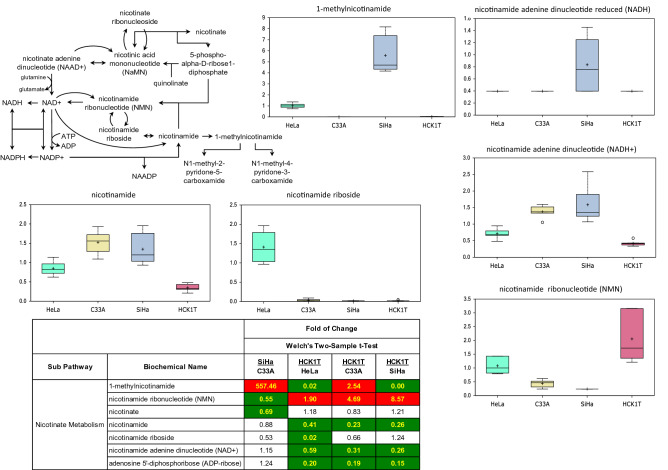
Metabolomic analysis of the differential nicotinate metabolism of the four cervical cell lines. Biochemicals displaying salient changes among the four cell types are shown as box plots. Each box plot provides a measure of scaled intensity, normalised by protein content. The nicotinate pathway map and a representative heat map, with the major biochemical changes is also shown. For the color key of the heat map, see the legend of Fig. 1.

Nicotinamide metabolism is central to energy production and aberrant metabolism is considered a hallmark of cancer. Consistent with this, we observed derangements in nicotinate metabolism in the different cancer cell lines, broadly resulting in differential alterations of intermediates within the nicotinamide pathway, including nicotinamide (Fig. 4; pathway map, heat map, box plots). Most strikingly, SiHa cells exhibited accretion of nicotinamide adenine dinucleotide (NAD), NADH, and methylated derivative 1-methylnicotinamide (Fig. 4; heat map, box plots). Furthermore, HCK1T cells exhibited higher concentrations of nicotinamide ribonucleotide (NMN), while HeLa cells possess increased nicotinamide riboside levels. Collectively, these disturbances likely provide required co-factors to fuel biosynthetic and epigenetic reactions to help confer growth advantage to transformed cells. Finally, SiHa cells displayed an increased cysteine/glutathione pathway activity.

### SiHa cells display increased cysteine/glutathione pathway activity

**Figure 5:**
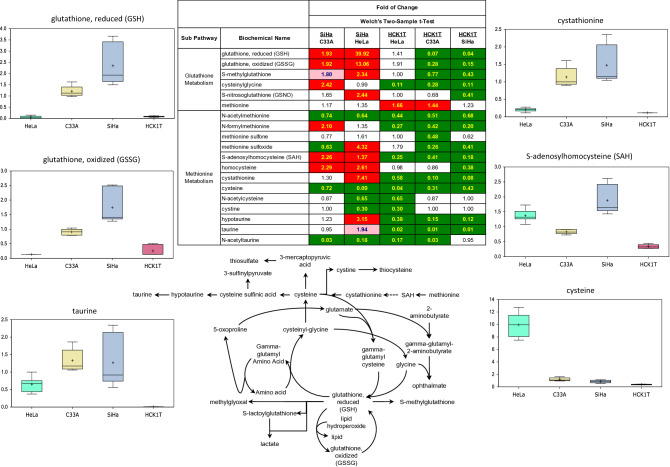
Metabolomic analysis of the cysteine/glutathione metabolism of the four cervical cell lines. SiHa and C33A cells displayed increased activity of numerous metabolites of this pathway. Each box plot provides a measure of scaled intensity, normalised by protein content. The cysteine/glutathione pathway map and a representative heat map, with the major biochemical changes is also shown. For the color key of the heat map, see the legend of Fig. 1.

Tumor cells frequently upregulate glutathione production to manage increased redox stress associated with higher proliferation rates. A primary antioxidant in cells is glutathione, which is synthesized from the precursor amino acids glutamate, cysteine, and glycine (Fig. 5; pathway map). Comparison of the four sample groups revealed that SiHa and C33A cells have similar profiles for metabolites in this pathway, showing increased levels of both reduced and oxidized glutathione, as well as an increase in many other metabolites associated with glutathione synthesis, such as S-methylglutathione, cysteinylglycine, and S-nitrosoglutathione (Fig. 5; heat map; box plots). While the increased level of oxidized glutathione may signal increased oxidative pressure, the fact that the reduced/oxidized ratio remains fairly constant, indicates that the transformed cells are managing the oxidative stress effectively. Consistent with changes to the glutathione pathway, we also observed parallel alterations in many components of tightly associated methionine and cysteine metabolism. Several intermediates, including methionine sulfoxide, S-adenosylmethionine, homocysteine, and cystathionine, all show evidence of accretion in SiHa and C33A cells, relative to both HeLa and HCK1T cells (Fig. 5; heat map, box plots). These metabolites also provide important precursors for hypotaurine and taurine, which have inherent antioxidant properties to help neutralize growth-driven oxidative stress (Fig. 5; heat map, pathway map, box plot). Interestingly, HeLa cells produce striking levels of cysteine, relative to other cell groups, which may represent an adaptation to neutralize reactive oxygen species (Fig. 5; box plot).

### Lipid metabolism

**Figure 6:**
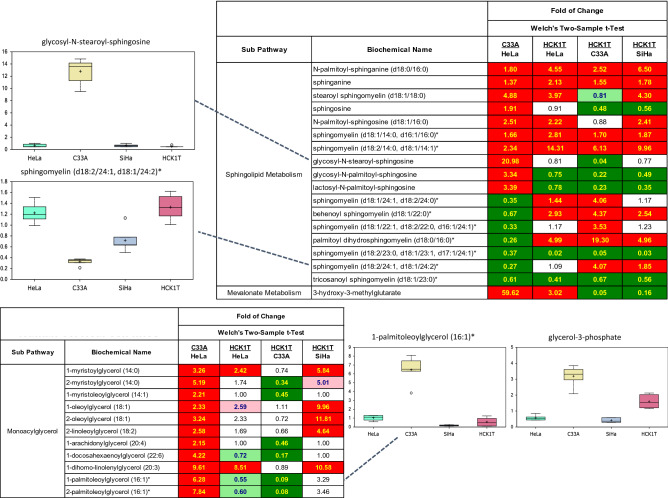
Metabolomic analysis of the lipid metabolism of the four cervical cell lines. Biochemicals displaying salient changes among the four cell types are shown as box plots. Each box plot provides a measure of scaled intensity, normalised by protein content. Two representative heat maps (upper and lower) with the major biochemical changes are shown. For the color key of the heat map, see the legend of Fig. 1.

Analysis of lipid levels again revealed distinct metabolic profiles among the four cancer cell lines. While there are robust changes in most lipid classes, this is particularly true of sphingolipids and monoacylglycerols (Fig. 6). Sphingolipids represent a major class of bioactive lipids that regulate a host of cellular functions, including proliferation, differentiation, migration, and apoptosis. Further, sphingosine can be phosphorylated to yield sphingosine-1-phosphate (S1P), a ligand for several G-protein-coupled receptors that regulate cell transformation, survival, migration, metastasis, and angiogenesis. The upper heat map and associated box plots in Fig. 6, illustrate the divergence of sphingolipid metabolism in these cells, with C33A exhibiting robustly higher concentrations of glycosylated sphingolipids such as glycosyl-N-stearoyl-sphingosine, but lower levels of sphingomyelin (d18:2/24:1/d18:1/24:2). C33A cells also exhibited a distinct monoacylglycerol profile (Fig. 6). Nearly all detected monoacylglycerols, including 1-myristoylglycerol (14:0), 1-oleolyglycerol (18:1), and 1-palmitoleoylglycerol (16:1), were elevated in C33A cells, relative to other cancer cells types (Fig. 6; lower heat map; associated box plot). Monoacylglycerols primarily derive from lipase activity on diacylglycerols, which also liberates a fatty acid. An increase in monoacylglycerols may suggest an increase in lipase activity. Consistent with this, we observed preferential accumulation of glycerol 3-phosphate, a precursor to phosphatidic acid and the diacylglycerols in C33A cells, possibly indicating higher diacylglycerol synthesis and subsequent degradation to produce monoacylglycerols, versus decreased synthesis (Fig. 6; box plot).

### C33A cells exhibit potentiation of arginine metabolism

**Figure 7:**
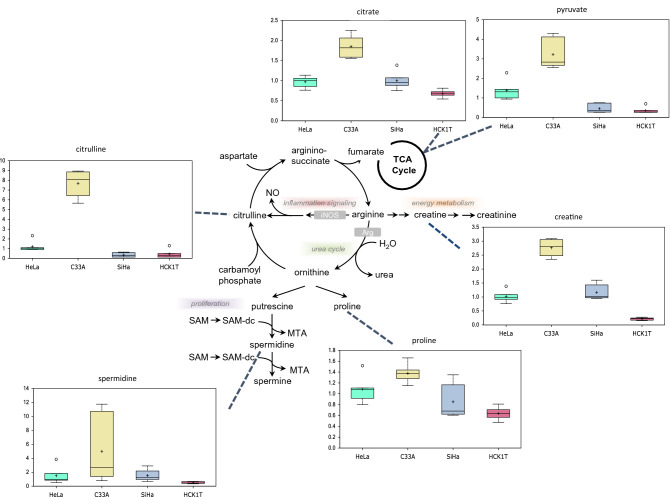
Metabolomic analysis of the arginine metabolism of the four cervical cell lines. C33A cells displayed potentiation of arginine metabolism. Biochemicals displaying salient changes among the four cell types are shown as box plots. Each box plot provides a measure of scaled intensity, normalised by protein content. The relevant pathway map of arginine metabolism is also shown.

The HPV-negative C33A cells displayed preferential accumulation of arginine pathway molecules, relative to the HPV-positive cancer cell groups, showing increases in citrulline and associated cycle components creatine and spermidine (Fig. 7; box plots). Elevations in the levels of the latter molecules often mark increased energy and proliferation, respectively. Consistent with this finding, these same cells exhibited higher levels of tricarboxylic acid (TCA) cycle intermediates citrate and pyruvate, indicating that arginine metabolism may help drive TCA cycle activity (Fig. 7; pathway map). This is especially intriguing, since C33A cells uniquely did not display features of Warburg metabolism that was evident in both SiHa and HeLa cells (Fig. 1), suggesting that C33A cells may utilize TCA-driven oxidative phosphorylation to help fuel growth.

### The normal cervical HCK1T cells exhibit elevation in dipeptide levels

**Figure 8:**
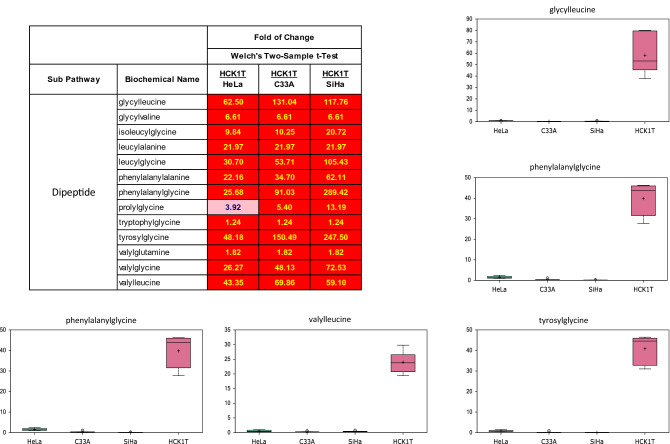
Documentation of elevated levels of many dipeptides exhibited exclusively by the normal HCK1T cells. Dipeptides levels are shown as box plots. Each box plot provides a measure of scaled intensity, normalised by protein content. A heat map, with the major biochemical changes is also shown. For the color key of the heat map, see the legend of Fig. 1.

To identify immortalization-associated changes independent from endogenous, cervical cancer-derived transformed cells, we sorted the dataset by significance of changes of normal HCK1T cells, relative to the other cells. Results revealed a large number of changes, with striking increases observed in dipeptide levels of HCK1T cells, including glycylleucine, leucylglycine, phenylalanylglycine, and tyrosylglycine (Fig. 8; heat map, box plots).

## Discussion

Metabolic reprograming represents one of the hallmarks of carcinogenesis^10^. During carcinogenesis, both cell metabolism and proliferation share common key pathways, which become deregulated to adjust to the nutritional and growth requirements of the cancer cells^1–3^. Therefore, delineating the aberrant pathways at the transcriptional, proteomic and metabolomics level, is anticipated to provide a global understanding of the mechanisms of human cancer, and can offer valuable insights for eventual development of rational diagnostic and therapeutic strategies. Cervical carcinogenesis is a multistage process following the carcinogenic infection of human papilloma virus (HPV) in virtually all cases of cancer^6^. Both HPV-positive and HPV-negative cervical cancers are associated with aberrant proliferation; however, the contribution of the individual oncogenic drivers remains elusive. In this study, we characterized the metabolomic profiles of four distinct informative cervical cell lines, in order to identify specific metabolic derangements –in the presence or absence of HPV, driving carcinogenesis. To this end, a normal cervical (HCK1T) cell line and three cervical cancer cell lines, one HPV-negative (C33A), and two HPV-positive (SiHa HPV16+ and HeLa HPV18+) were used. This study represents a rational extension of our recent studies on cervical cancer at the transcriptomic^11^ and the proteomic level^12^, utilizing either cervical cancer tissue specimens or the secretome of the four cell lines, respectively. To our knowledge, the present study represents the first report focusing on the metabolomics signatures of cervical cancer cell lines, both HPV-positive and HPV-negative, as well as normal.

The dataset documented a total of 462 biochemicals, of which 248 to 326 exhibited statistically significant differences (*P* ≤ 0.05) among the four cell lines, while Random Forests analysis identified molecules distinguishing the four cell lines, indicating that the individual cell lines exhibited highly robust and distinct global biochemical profiles.

Analysis of the carbohydrate metabolism revealed that both HPV-positive cells exhibited the Warburg effect. This is consistent with a recent report showing that HPV E6 oncoprotein promotes Warburg metabolism by stabilizing the expression of the glycolytic mediator HIF-1α^13^. It is interesting that recently, the metabolomics profile of γ-irradiated human hepatoma and muscle cells exhibited also metabolic changes consistent with the Warburg effect^14^, with potential shunting of glucose through aldose reductase in the polyol pathway, and consumption of reduced glutathione due to γ-irradiation. Furthermore, both these HPV-positive cell lines exhibited a very active pentose phosphate pathway (PPP). This may serve to fuel 5-phosphoribosyl diphosphate (PRPP) production, an important intermediate for purine synthesis, particularly in SiHa cells.

It is important to emphasize the fact that PPP plays a significant role in maintaining a high cell proliferation rate, and thus represents the main cellular metabolic activity for the development of the so-called tumor drug resistance^15^, possibly mediated via metabolic reprogramming observed in cancer cells, as exemplified by the well-described cisplatin resistance^16^. The mechanism by which this resistance is mediated, has been found to arise by the overexpression and high enzymatic activity of particular enzymes of the pathway, such as glucose-6-phosphate dehydrogenase, 6-phosphogluconate dehydrogenase and transketolase^15^. Eventually, silencing or downregulation of their activity by putative inhibitors via novel drug delivery systems targeting selectively cancer cells, could potentially restore cisplatin sensitivity^17^. However, many aspects of the cisplatin resistance mechanisms are still elusive and warrant further studies. Additionally, the altered expression of selected miRNAs in resistant cell lines compared to their parental HeLa cell line, suggests that drug resistance is also associated with distinct miRNAs, which can constitute alternative targets for the treatment of cervical cancer^18^. It is conceivable that in our study, the documented rewiring of the PPP in the two HPV-positive cell lines might reflect the putative aberrant expression of the above enzymatic and miRNA activities, without necessarily leading to drug resistance. To precisely address this question in the future, a thorough analysis of the enzymes involved in the PPP along with the candidate miRNAs is required, followed by studies of the potential effects of the corresponding specific PPP inhibitors.

Furthermore, the PPP pathway plays also a crucial role in the metabolic coordination of glycolysis, via production of nicotinamide adenine dinucleotide phosphate (NADPH), which is not only required for biosynthesis of lipids, but is also a crucial antioxidant for neutralization of reactive oxygen species (ROS) produced during rapid proliferation of cancer cells to help maintain redox homeostasis. Due to a lack of associated changes in reporters for TCA cycle and glycolysis in the HPV-negative cells, the reduced levels of the PPP intermediates in these cells, may represent alternate utilization of carbohydrate oxidation for glycerol production, perhaps to provide backbone for glycerolipid synthesis.

Regarding the analysis of nucleic acid metabolism, our study revealed a unique pattern for each cell line. Specifically, the HPV-positive cells displayed high levels of purine derivatives, perhaps to facilitate nucleic acid production and support growth. Instead, normal HCK1T cells exhibited preference for adenosine metabolism, while HPV-negative C33A cells uniquely exhibited high concentrations of cytidine and cytidine 5′-monophosphate (5′-CMP), through a novel mechanism which remains to be determined. However, since the documented levels of cytidine 5′-phosphocholine –which is required for lipid biosynthesis by cytidine, do not change in proportion to cytidine, further work is required to elucidate the role of cytidine in C33A growth. The low levels of β-alanine in HCK1T cells, can be explained by the recent data establishing a link between the relative abundance of β-alanine along with pantothenic acid and glycerophosphoglycerol, to the glycolytic activity of cancer cell lines, as measured by lactate production^19^.

SiHa cells displayed characteristically high levels of NAD, NADH and methylated 1-methylnicotinamide, in the context of nicotinamide metabolism. Elevation of these metabolites could not only drive increased energy demands, but also support a host of proteome and genome modifications to maintain high growth rates. Further, recent studies provide compelling evidence that elevations of the enzyme responsible for methylation of nicotinamide to 1-methylnicotinamide (nicotinamide *N*-methyltransferase) results in induction of mitochondrial activity and ATP synthesis and may play a key role of sustained SiHa growth^20^. While NAD/NADH is central to energy homeostasis, it is also a required cofactor for the sirtuin family of protein deacetylases. Thus, elevation of these metabolites in HPV-positive cells, could not only drive increased energy demands, but also support a host of proteome and genome modifications to maintain high growth rates.

Interestingly, the SiHa and the C33A cancer cell lines, exhibited similar profiles for metabolites of the cysteine/glutathione pathway, suggesting that the tumor-derived transformed cervical cancer cells, regardless of the presence or absence of the HPV, appear to effectively and differentially deploy glutathione metabolism to help maintain favorable redox environment.

The divergence of sphingolipid metabolism among the four cell lines, and particularly of the C33A cells exhibiting significantly higher levels of glycosylated sphingolipids associated with a preferential accumulation of glycerol-3-phosphate, a precursor of phosphatidic acid and the diacylglycerols, warrants additional work to assess the relative roles of these lipids in membrane synthesis and fluidity, which may influence invasive/metastatic potential in cancer cells, signaling and energy production via lipid oxidation that give rise to distinct growth adaptations for each cell type.

A preferential accumulation of arginine pathway molecules was documented in C33A cells associated with high levels of TCA intermediates, as compared to the other cervical cancer cell types, although these HPV-negative cells did not exhibit Warburg metabolism, which implies that the TCA utilization is intended for fuel growth. Recent work suggests that p53 plays a central role in cellular bioenergetics by regulating the switch between oxidative phosphorylation and glycolysis, depending on oxygen tension^21^. Thus, mutant p53 function may help drive the observed bioenergetic output in C33A cells. Finally, the arginine circuit also influences protein turnover via proline metabolism. C33A cells show evidence of elevated proline levels**,** possibly indicating that a portion of arginine metabolism may be directed to support protein turnover, perhaps to be utilized as substrate for other anabolic reactions^22^.

Finally, our study documented for the first time the presence of striking increases in dipeptide levels in normal HCK1T cells. The uptake of small peptides by the Slc15A family of oligo/dipeptide transporters, provides an effective and energy-saving intracellular source of amino acids. This may represent a unique pathway deployed by HCK1T cells to provide alternate sources of components to increase biomass and fuel growth. Indeed, this has been observed in transformed hematopoietic stem cells (HSC), where increases in dipeptide levels are thought to be required to sustain stem cell maintenance^23^. Most importantly, this survival mechanism does not appear to operate routinely in normal HSCs and may provide a therapeutic opportunity to slow or eradicate the malignant clone(s). Alternatively, dipeptides also may play a role in the management of oxidative and glycation stress and HCK1T cells may co-opt these biochemicals to help neutralize oxidative stress.

In conclusion, our study documented for the first time the effects of the adaptation of HPV-positive and HPV-negative transformed cells by rewiring metabolic pathways to fuel unrestrained growth and exhibiting differential deregulation of several cellular processes, resulting in distinct HPV-specific metabolic profiles, and including molecules in the carbohydrate, nucleotide, lipid, and glutathione pathways. Interestingly, the present findings are consistent and fully support our recent high resolution proteomic data of these informative cell lines^24^, where the prominent and significant pathways found to be deregulated between the cervical cancer and the normal cell line, were those of the metabolism of proteins, metabolism of nucleotides, unwinding of DNA, glycolysis, gluconeogenesis apoptosis and oxidative stress-induced senescence. These pathways actually reflect the malignant phenotype of the cancer cells with high metabolic requirements, increased cell turnover and damage control mechanisms. Normal HCK1T cells were unique in many ways, including relative lack of glutathione, adapting instead to utilize dipeptide metabolism to provide sources of organic acids to maintain biomass and help neutralize growth-driven oxidative stress.

Collectively, these results reflect highly dynamic differences among the cellular groups, consistent with the recently established notion that cancer heterogeneity is not compatible with one unique cancer cell metabolic map^25,26^. Therefore, these data provide the impetus to further elucidate both unique and common aspects of perturbed growth profiles of human cervical cancer cells.

## Methods

### Cell lines, culture conditions and sample preparation

SiHa, HeLa and C33A cells were purchased from ATCC and cultured in DMEM, supplemented with 10% FBS, 1% P/S (supplied by Gibco-Invitrogen, Waltham, MA, USA) at 37 °C and 5% CO_2_, as previously described by us^12^. ΗCK1T cells were a kind gift of Dr Tohru Kiyono^8,9^, and were cultured as proposed^8,9^ in Defined Keratinocyte Serum-Free Medium (Gibco-Invitrogen), supplemented with 5 ng/ml EGF (Epidermal Growth Factor; Gibco-Invitrogen) and 50 μg/ml of BPE (Bovine Pituitary Extract; Gibco-Invitrogen). For harvesting, following the removal of the growth medium, ~ 3 × 10^6^ of attached cells were washed once with PBS, and the bottom of the flask was lightly scraped using a cell scraper in 1 ml solution of 80% ice-cold methanol:20% water, and the detached cells were suspended in PBS. Cell handling was performed on ice. The samples were further centrifuged at 1200 rpm, the supernatant was removed and the cellular pellets (~ 100 μl in size) from six biological replicates for each line, were flash frozen and stored at –80 °C until further use. Extraction of samples were prepared using the automated MicroLab STAR system from Hamilton Company (Reno, NV, USA). Recovery standards were added prior to the first step in the extraction process for quality control (QC) purposes. To remove protein, dissociate small molecules bound to protein or trapped in the precipitated protein matrix, and to recover chemically diverse metabolites, proteins were precipitated with methanol under vigorous shaking for 2 min (Glen Mills GenoGrinder 2000, Lebanon, NJ, USA) followed by centrifugation. The resulting extract was divided into five fractions: two for analysis by two separate reverse phase (RP)/ultra performance liquid chromatography-tandem mass spectrometry (UPLC-MS/MS) methods with positive ion mode electrospray ionization (ESI), one for analysis by RP/UPLC-MS/MS with negative ion mode ESI, one for analysis by hydrophilic interaction liquid chromatography (HILIC)/UPLC-MS/MS with negative ion mode ESI, and one sample was reserved for backup^27^. Samples were placed briefly on a TurboVap (Zymark, Hopkinton, MA, USA) to remove the organic solvent. The sample extracts were stored overnight under liquid nitrogen before preparation for analysis.

### Quality assurance and quality control

Several types of controls were analyzed in concert with the experimental samples: a pooled matrix sample generated by taking a small volume of each experimental sample; extracted water samples served as process blanks; and a cocktail of QC standards that were carefully chosen not to interfere with the measurement of endogenous compounds were spiked into every analyzed sample, allowed instrument performance monitoring and aided chromatographic alignment. Instrument variability was determined by calculating the median relative standard deviation (RSD) for the standards that were added to each sample prior to injection into the mass spectrometers^28^. Overall process variability was determined by calculating the median RSD for all endogenous metabolites (i.e., non-instrument standards) present in 100% of the pooled matrix samples. Experimental samples were randomized across the platform run with QC samples spaced evenly among the injection.

### Ultrahigh performance liquid chromatography-tandem mass spectroscopy (UPLC-MS/MS)

All methods utilized a Waters (Milford, MA, USA) ACQUITY ultra-performance liquid chromatography (UPLC) and a Thermo Scientific (Waltham, MA, USA) Q-Exactive high resolution/accurate mass spectrometer interfaced with a heated electrospray ionization (HESI-II) source and Orbitrap mass analyzer operated at 35,000 mass resolution^27^. The sample extract was dried and then reconstituted in solvents compatible to each of the four methods. Each reconstitution solvent contained a series of standards at fixed concentrations to ensure injection and chromatographic consistency. One aliquot was analyzed using acidic positive ion conditions, chromatographically optimized for more hydrophilic compounds. Specifically, the extract was gradient eluted from a C18 column (Waters UPLC BEH C18-2.1 × 100 mm, 1.7 µm) using water and methanol, containing 0.05% perfluoropentanoic acid (PFPA) and 0.1% formic acid (FA). Another aliquot was also analyzed using acidic positive ion conditions; however, it was chromatographically optimized for more hydrophobic compounds. In this method, the extract was gradient eluted from the same aforementioned C18 column using methanol, acetonitrile, water, 0.05% PFPA and 0.01% FA and was operated at an overall higher organic content. Another aliquot was analyzed using basic negative ion optimized conditions using a separate dedicated C18 column. The basic extracts were gradient eluted from the column using methanol and water, however with 6.5 mM ammonium bicarbonate at pH 8. The fourth aliquot was analyzed via negative ionization following elution from a HILIC column (Waters UPLC BEH Amide 2.1 × 150 mm, 1.7 µm) using a gradient consisting of water and acetonitrile with 10 mM ammonium formate, pH 10.8. The MS analysis alternated between MS and data-dependent MS^n^ scans using dynamic exclusion. The scan range varied slightly between methods but covered 70–1000 m/z. Raw data files were archived and extracted as described below.

### Metabolite quantification and data normalization

Peaks were quantified using area-under-the-curve. Values were normalized in terms of raw area counts. For a single day run, this was equivalent to the raw data. Each biochemical in OrigScale was rescaled to set the median equal to 1. Then, missing values were imputed with the minimum. For studies spanning multiple days, a data normalization step was performed to correct variation resulting from instrument inter-day tuning differences. Essentially, each compound was corrected in run-day blocks by registering the medians to equal one (1.00) and normalizing each data point proportionately (termed the “block correction”). For studies that did not require more than one day of analysis, no normalization was necessary, other than for purposes of data visualization. In certain instances, biochemical data have been normalized to an additional factor, such as total protein, determined by Bradford assay as previously described by us^29^, to account for differences in metabolite levels due to differences in the amount of material present in each sample. Collectively, all interpreted data were normalized based on protein content, while all statistics performed were on natural log transformed data, given the well-documented right skew of metabolomics data. Finally, the Welch’s two-sample *t*-test was used to identify biochemicals that differed significantly between the four cell lines.

### Data extraction and compound identification

Raw data was extracted, peak-identified and QC processed using Metabolon’s hardware and software^27,30^. Compounds were identified by comparison to library entries of purified standards or recurrent unknown entities, utilizing a maintained library based on authenticated standards that contains the retention time/index (RI), mass to charge ratio (m/z), and chromatographic data (including MS/MS spectral data) on all molecules present in the library^30,31^. The biochemical identifications were based on three criteria: retention index within a narrow RI window of the proposed identification, accurate mass match to the library ± 10 ppm, and the MS/MS forward and reverse scores between the experimental data and authentic standards. The MS/MS scores were based on a comparison of the ions present in the experimental spectrum to the ions present in the library spectrum.

### Bioinformatics

The informatics system consisted of four major components, the Laboratory Information Management System (LIMS), the data extraction and peak-identification software, data processing tools for QC and compound identification, and a collection of information interpretation and visualization tools for use by data analysts^32^. The hardware and software foundations for these informatics components were the LAN backbone, and a database server running Oracle 10.2.0.1 Enterprise Edition.

### Statistical analysis

The Welch’s two-sample *t*-test was used to identify biochemicals that differed significantly between experimental groups. For statistical significance testing, *p*-values < 0.05, were considered to be significant. An estimate of the false discovery rate (*q*-value) was also calculated to take into account the multiple comparisons that normally occur in metabolomic-based studies, whose *p*-value (*P* < 0.05) was considered as a cut-off for significance^33^. Random Forests analysis^34^ was used to bin individual samples in groups based on their metabolite similarities and differences and to identify metabolites that differentiated among the cell lines. Both principal component analysis (PCA) and hierarchical clustering were used as unsupervised methods for grouping the data, as previously described by us^35^.

## Supplementary Information

Extended Data legends.

Extended Data Figure 1.

Extended Data Figure 2.

Extended Data Figure 3.

Extended Data Table 1.

Extended Data Table 2.
