# Supplementary material for: Metabolic rewiring is associated with HPV-specific profiles in cervical cancer cell lines
Source: Sci Rep. 2021 Sep 6;11:17718. doi: 10.1038/s41598-021-96038-8 (PMC8421399; doi:10.1038/s41598-021-96038-8)
Supplement: Supplementary file 1 — Extended Data legends. [file 41598_2021_96038_MOESM1_ESM.docx]

**Title Page**

**Metabolic rewiring is associated with HPV-specific profiles in cervical cancer cell lines**

**Kalliopi I. Pappa**^1,2^**, George Daskalakis**^2^**, and Nicholas P. Anagnou**^1,3,*^

^1^Cell and Gene Therapy Laboratory, Biomedical Research Foundation of the Academy of Athens (BRFAA), Athens, Greece

^2^First Department of Obstetrics and Gynecology, National and Kapodistrian University of Athens School of Medicine, Athens, Greece

^3^Laboratory of Biology, National and Kapodistrian University of Athens School of Medicine, Athens, Greece

**Extended Data legends**

**Extended Data Fig. 1. Principal component analysis (PCA) of the metabolomics profiles of the three cervical cell lines (HeLa, SiHa and C33A) and the normal control HCK1T cells, with each cell line analyzed in six biological replicates**. Principal component analysis in three axes, depicts the clear separation of the normal and the cancer cell lines in four tightly group clusters.

**Extended Data Fig. 2. Hierarchical clustering analysis of the data set of metabolomics profiles of the four cell lines, with each cell line analyzed in six biological replicates**. A strong separation of each cell segregating into four distinct groups is shown, using Array Studio v7.0 (OmicSoft/QIAGEN Bioinformatics) and employing Euclidean distance.

**Extended Data Fig. 3. Random Forests analysis of the data set of metabolomics profiles of the four cell lines, with each cell line analyzed in six biological replicates, to identify metabolites that differentiated among the four cellular groups**. The analysis yielded a predictive accuracy of 100%, compared to 25% by random chance alone, suggesting that the biochemical differences among the sample groups were highly pronounced. The top 30 biochemicals are shown, based on their importance in separating the metabolic profiles for all groups. Molecules that powerfully distinguished the four cell lines include sugars (ribose, sedohepulose-7-phosphate, and UDP-glucose), lipids [e.g. palmitoyl dihydrosphingomyelin (d18:0/16:0)*, 1-steroyl-GPE (18:0)], and nucleotide breakdown products (hypoxanthine, xanthosine).

**Extended Data Tables**

**Extended Data Table 1.** Summary of the biochemicals that achieved either statistical significance (*P* ≤ 0.05) or approached significance (0.05 < *P* < 0.10), as derived from the six-paired comparisons among of the four cell lines.

Red arrows and numbers in red indicate biochemicals that displayed upregulation, while green arrows and green numbers indicate biochemicals that exhibited downregulation.

**Extended Data Table 2**. Complete lists of data and statistical analysis of all the identified biochemicals in the four cervical cell lines, each line analyzed in six biological replicates. Explanation of the lists is provided in Spreadsheet 1 (Explanation); list of all biochemical tested is shown in Spreadsheets 2-4 (OrigScale, ScaledImpData and ProNormImpData); box plots per pathway and per biochemical are shown in Spreadsheets 5 and 7, respectively, and Heat maps of statistically significant biochemicals profiled in this study, are shown in Spreadsheet 6 (Pathway Heat Map).
