# Supplementary figures and images for: Metabolic rewiring is associated with HPV-specific profiles in cervical cancer cell lines
Source: Sci Rep. 2021 Sep 6;11:17718. doi: 10.1038/s41598-021-96038-8 (PMC8421399; doi:10.1038/s41598-021-96038-8)

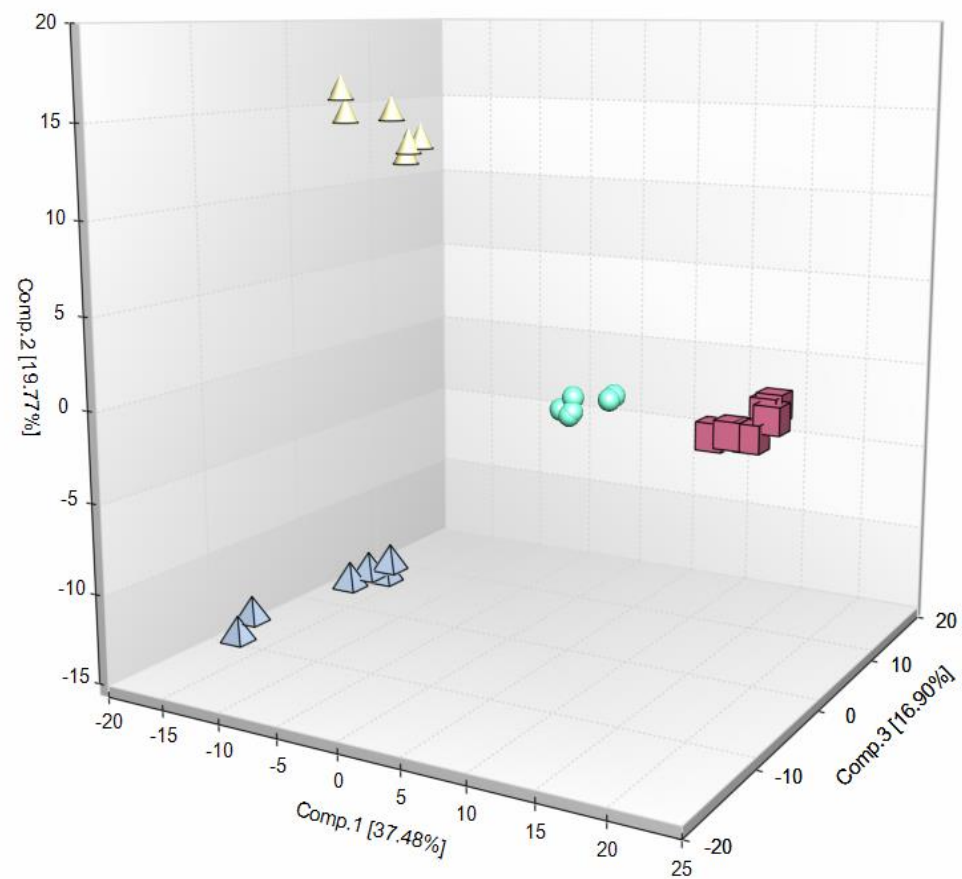

Color by Group

● HeLa

● C33A

● SiHa

● HCK1T

Shape by Group

● HeLa

▲ C33A

▲ SiHa

■ HCK1T

Supplement: Supplementary file 2 — Extended Data Figure 1. [file 41598_2021_96038_MOESM2_ESM.pdf]

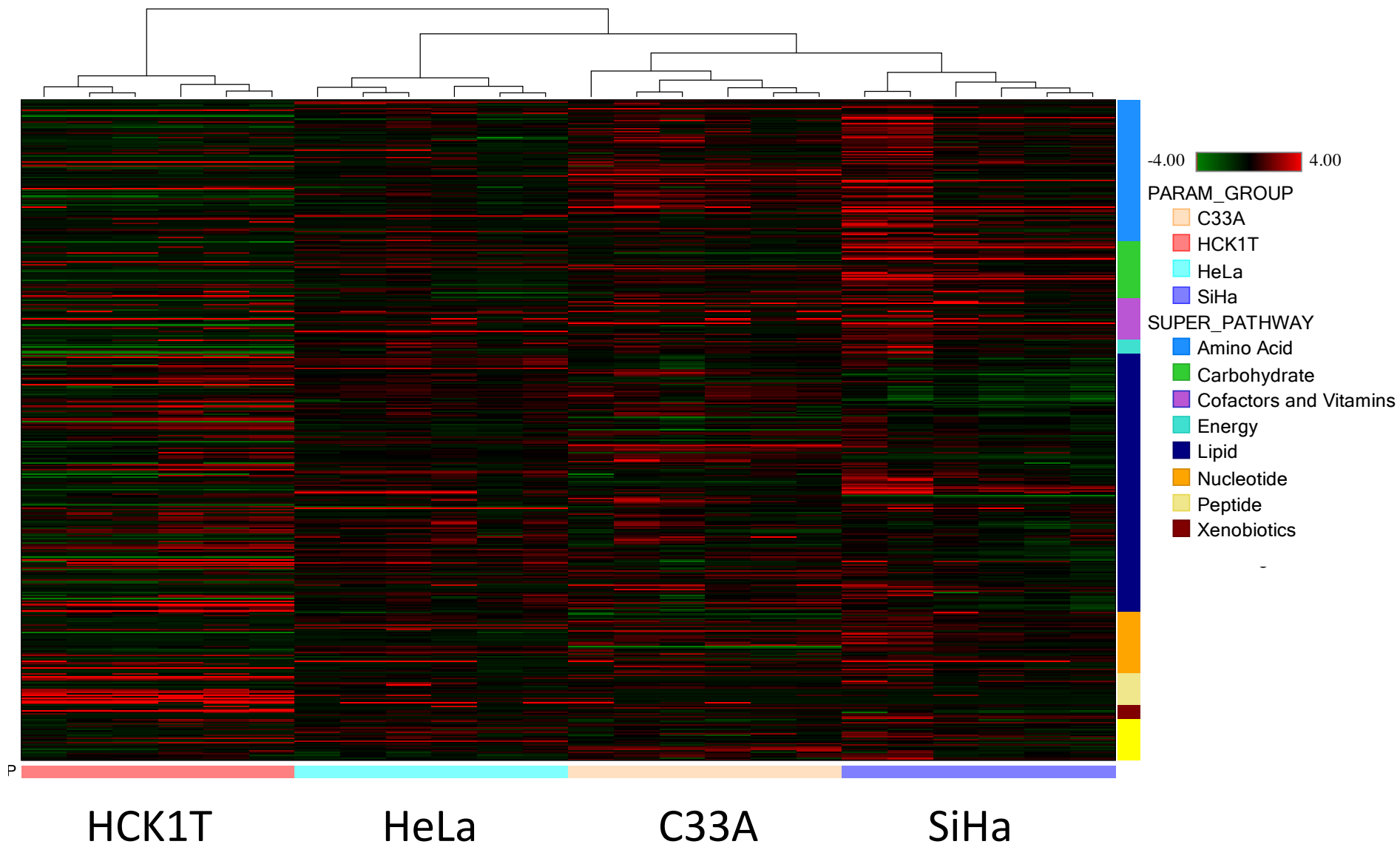

Extended Data Fig. 2

Supplement: Supplementary file 3 — Extended Data Figure 2. [file 41598_2021_96038_MOESM3_ESM.pdf]
