# Supplementary material for: Metabolic rewiring is associated with HPV-specific profiles in cervical cancer cell lines
Source: Sci Rep. 2021 Sep 6;11:17718. doi: 10.1038/s41598-021-96038-8 (PMC8421399; doi:10.1038/s41598-021-96038-8)
Supplement: Supplementary file 4 — Extended Data Figure 3. [file 41598_2021_96038_MOESM4_ESM.pdf]

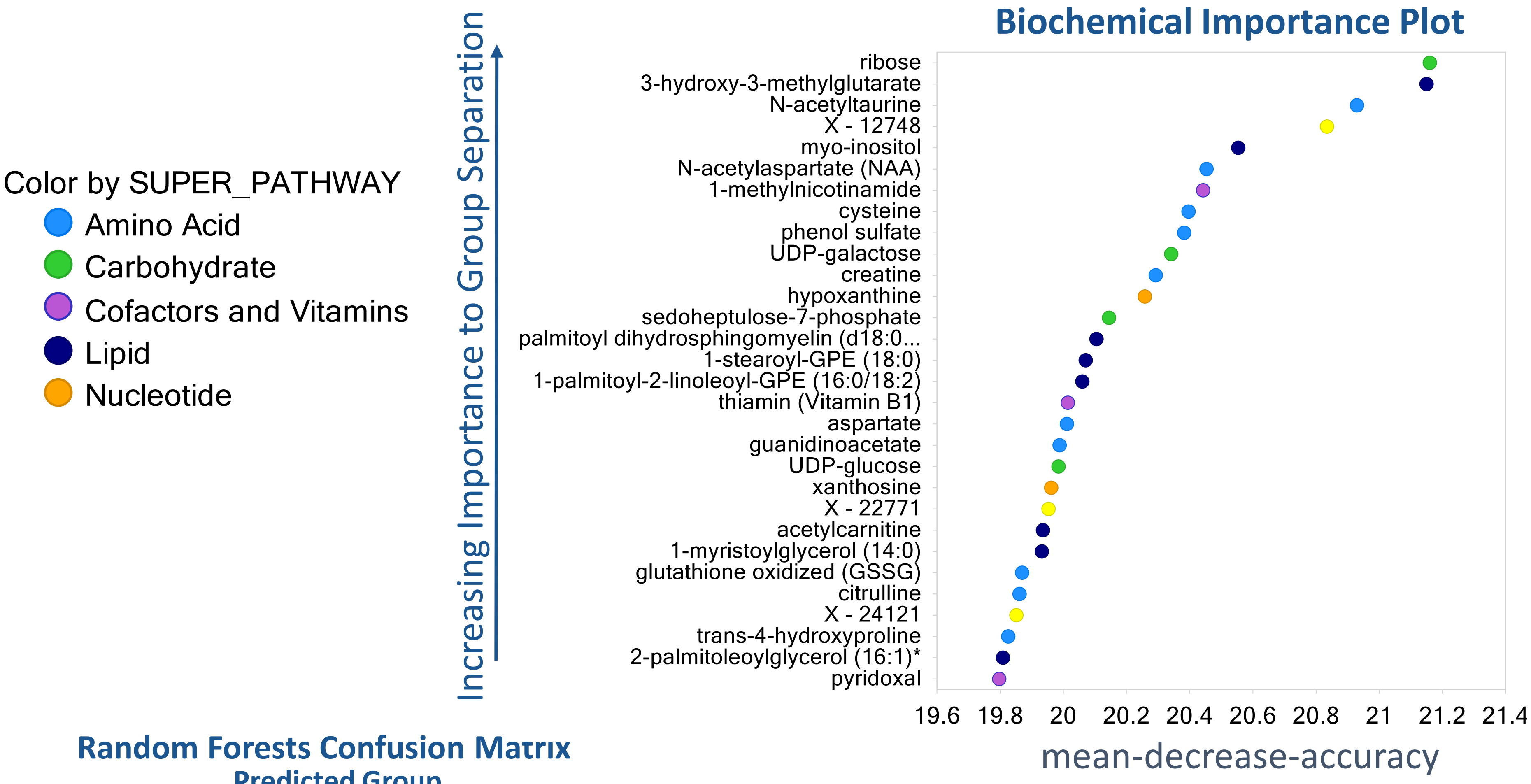

Random Forests Confusion Matrix

Predicted Group

Actual Group

	C33A	HCK1T	HeLa	SiHa	Class Error
C33A	6	0	0	0	0
HCK1T	0	6	0	0	0
HeLa	0	0	6	0	0
SiHa	0	0	0	6	0
Predictive Accuracy = 100%					

Extended Data Fig. 3
