# Supplementary material for: Metabolic rewiring is associated with HPV-specific profiles in cervical cancer cell lines
Source: Sci Rep. 2021 Sep 6;11:17718. doi: 10.1038/s41598-021-96038-8 (PMC8421399; doi:10.1038/s41598-021-96038-8)
Supplement: Supplementary file 5 — Extended Data Table 1. [file 41598_2021_96038_MOESM5_ESM.docx]

**Extended Data Table 1**


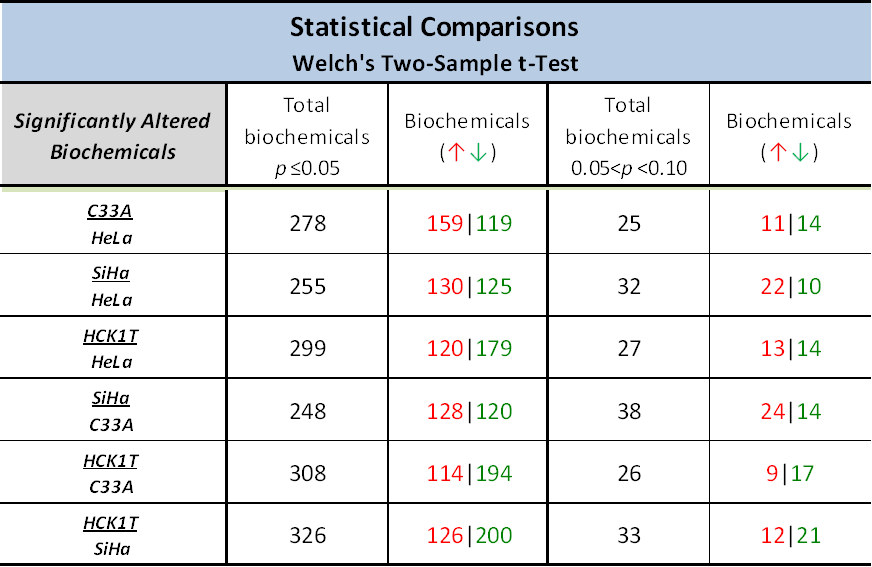


P ≤ 0.05

0.05 < P < 0.10

**Extended Data Table 1.** Summary of the biochemicals that achieved either statistical significance (*P* ≤ 0.05) or approached significance (0.05 < *P* < 0.10), as derived from the six-paired comparisons among of the four cell lines.

Red arrows and numbers in red indicate biochemicals that displayed upregulation, while green arrows and green numbers indicate biochemicals that exhibited downregulation.
